# Supplementary figures and images for: Comparing long-term value creation after biotech and non-biotech IPOs, 1997–2016
Source: PLoS One. 2021 Jan 6;16(1):e0243813. doi: 10.1371/journal.pone.0243813 (PMC7787373; doi:10.1371/journal.pone.0243813)

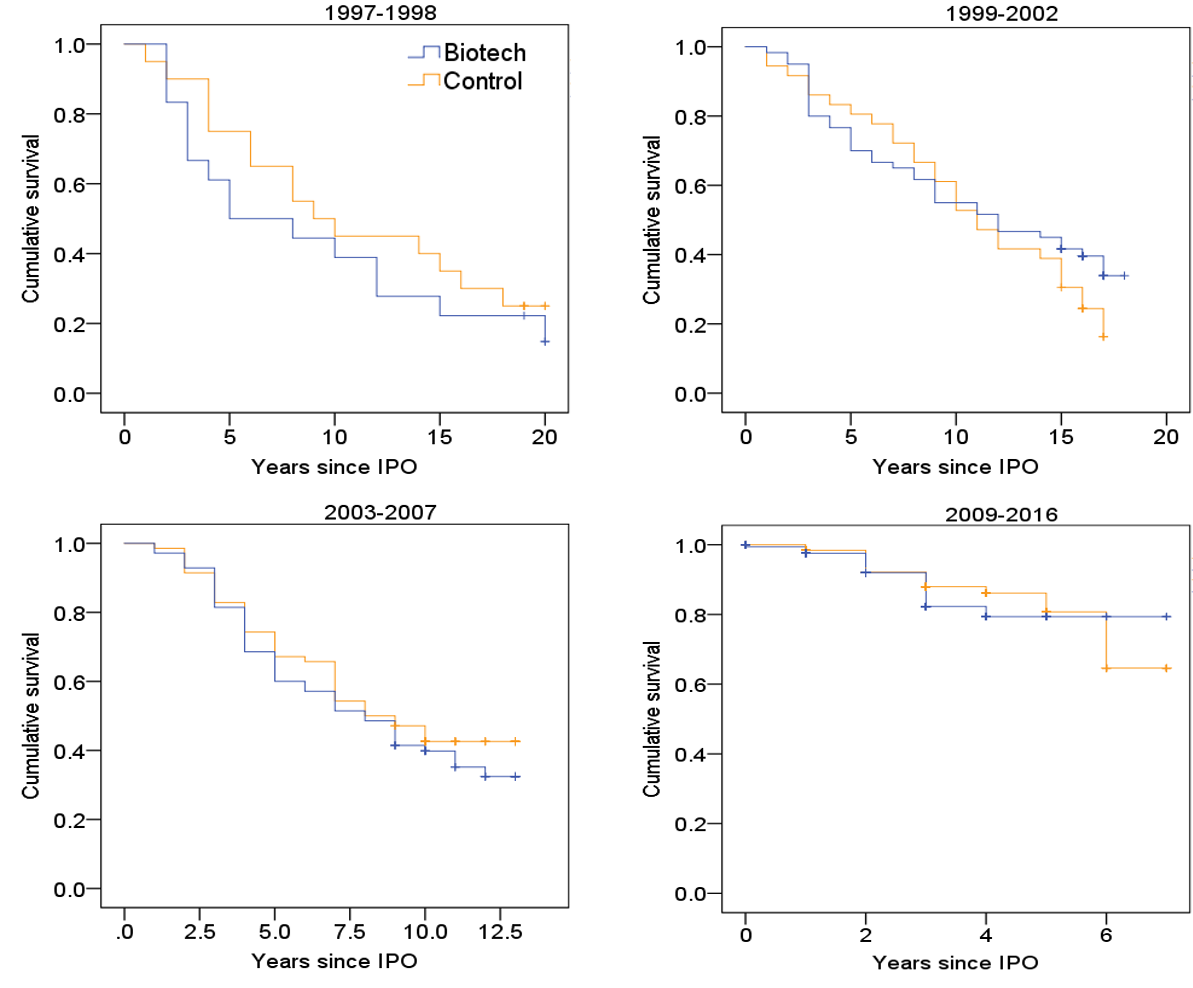

Supplement: S1 Fig — (TIF) [file pone.0243813.s001.tif]
