# Supplementary material for: Comparing long-term value creation after biotech and non-biotech IPOs, 1997–2016
Source: PLoS One. 2021 Jan 6;16(1):e0243813. doi: 10.1371/journal.pone.0243813 (PMC7787373; doi:10.1371/journal.pone.0243813)
Supplement: Attachment — Submitted filename: REVIEWERS COMMENTS AND RESPONSE.docx [file pone.0243813.s003.docx]

September 23, 2020

Alejandro Raul Hernandez Montoya, PhD
Academic Editor
PLOS ONE

Re:	PONE-D-20-04037
Comparing long-term value creation after biotech and non-biotech IPOs, 1997-2016

Dear Dr. Montoya,

Thank you very much for your review of our manuscript. We recognize that these are difficult times, and that this type of highly interdisciplinary work spanning biotechnology and business disciplines is difficult to review.

We appreciate the comments of your reviewers, and are resubmitting a manuscript that has been revised to address their suggestions. As noted in your email, we are uploading a marked-up copy of the manuscript with Track Changes, an unmarked version without Track Changes, and this rebuttal letter. In this letter, the reviewer’s comments are copied in **BOLD**. Our responses are indented in plain font and begin on pg. 2 of this document. The PACE system was used to generate 300 dpi resolution figures.

There are no changes to our financial disclosures.

There are no laboratory protocols associated with this work.

**Comments to the Author**

**1. Is the manuscript technically sound, and do the data support the conclusions?**

**The manuscript must describe a technically sound piece of scientific research with data that supports the conclusions. Experiments must have been conducted rigorously, with appropriate controls, replication, and sample sizes. The conclusions must be drawn appropriately based on the data presented.**

**Reviewer #1: Yes**

**Reviewer #2: Partly**

________________________________________

**2. Has the statistical analysis been performed appropriately and rigorously?**

**Reviewer #1: I Don't Know**

**Reviewer #2: Yes**

________________________________________

**3. Have the authors made all data underlying the findings in their manuscript fully available?**

**The PLOS Data policy requires authors to make all data underlying the findings described in their manuscript fully available without restriction, with rare exception (please refer to the Data Availability Statement in the manuscript PDF file). The data should be provided as part of the manuscript or its supporting information, or deposited to a public repository. For example, in addition to summary statistics, the data points behind means, medians and variance measures should be available. If there are restrictions on publicly sharing data—e.g. participant privacy or use of data from a third party—those must be specified.**

**Reviewer #1: Yes**

**Reviewer #2: Yes**

________________________________________

**4. Is the manuscript presented in an intelligible fashion and written in standard English?**

**PLOS ONE does not copyedit accepted manuscripts, so the language in submitted articles must be clear, correct, and unambiguous. Any typographical or grammatical errors should be corrected at revision, so please note any specific errors here.**

**Reviewer #1: Yes**

**Reviewer #2: Yes**

________________________________________

**5. Review Comments to the Author**

**Please use the space provided to explain your answers to the questions above. You may also include additional comments for the author, including concerns about dual publication, research ethics, or publication ethics. (Please upload your review as an attachment if it exceeds 20,000 characters)**

**Reviewer #1: As far as I understand the approach the authors too a relevant question is new and very interesting**.

**On the one hand the fact that pairs are chosen is relevant to this approach and probably increases significance of the approach, but the criteria of the choice, adequately discussed in the article, obviously leave room for arbitrariness. On the other hand, the very fact that pairs with comparable dates and other conditions have been chosen, gives the entire study a strong background of credibility. Yet credibility and statistical significant are not the same, but I personally weigh credibility as a very important factor. This is the basis for my judgement that the data support the conclusions but that I do not know if the statistics have been performed rigorously, yet I think they have been performed appropriately in the sense that the best was done with the data available.**

Thank you for your comments about the challenge of knowing whether statistical tests are “credible” and meaningful, as well as quantitatively significant. In formal, statistical language, the statistical analysis simply estimates the probability of observing the data we described, assuming the null hypothesis that there is no difference between the BIOTECH and paired CONTROL datasets. In our experimental design, the paired companies were selected to have comparable IPO dates as a control for general market conditions, which is known to be a major factor in any study of company stock performance.

We would note that treating any particular p value as “significant” is arbitrary. Following the recommendations of the American Statistical Association, this manuscript reports the calculated p-values themselves, and refrains from designating any specific value as significant or not significant. (The ASA Statement on p-Values: Context, Process, and Purpose, <https://amstat.tandfonline.com/doi/full/10.1080/00031305.2016.1154108>)

**I wish to emphasize that the authors plainly state what they do and how they obtain their conclusions, allowing the reader to draw his own conclusions, if he so wishes!**

**As for the relevance, I believe that the present crisis enhances the relevance of this paper, as biotech firms play a really important role, and to know that the high risk assigned to biotech even in venture capital circles may well be exaggerated.**

**Summarizing I wish to say, that I strongly support the publication of this paper in its present form, as it is innovative, clearly written and timely!**

**Reviewer #2: This paper's objective, to compare the financial performance of BIOTECH companies with the performance of NON-BIOTECH companies, is very interesting. However, there are needed the following points: (1) more rigorous explanation on the methodology techniques used within the research (especially on the Kaplan-Meier time-to-event analysis);**

As requested, we have added a section to the Methods section detailing the Kaplan-Meier time-to-event analysis on pg. 19 and added a short, non-technical description of the method in the text on pg. 8. We also added an explanation of the use of the Kolmogorov-Smirnov and Bonferroni correction on pgs. 19 and 20, respectively.

**(2) to provide significance information about the arguments of choosing the financial variables (annual revenue, R&D expense, EBITDA, net income or loss), considering the differences in the business architecture of BIOTECH and NON-BIOTECH companies; and**

We have added a section to Results describing each of these variables describing these variables and their relevance on pg. 9. The question about the relevance of accounting metrics given the different architecture of BIOTECH and NON-BIOTECH companies is important. We would note that this analysis focuses explicitly on financial metrics based on data prepared in accordance with U.S. US GAAP standards (Generally Accepted Accounting Practice). These standards are designed explicitly to provide comparability and consistency across industrial sectors and between companies, and are used by investors as benchmarks to guide investments in companies with different architectures. We would not claim that these metrics are perfect comparators, and, as noted in the text, in selecting the paired controls, we eliminated companies in certain areas that provided particularly problematic comparisons.

**(3) the reasons of the results instead of just analysis. What are the implications of the results? A significant part of the research is only a descriptive analysis**.

We have extensively revised a portion of the discussion to address the implications of the results, while carefully staying away from any inferences of causality from the statistical analysis. Much of this revision involved consolidating some of the comments originally scattered through the Results section into a more coherent Discussion starting on pg. 14.

**The topic of this manuscript falls within the scope of PLOS ONE.**
